# Regulation of intracellular activity of *N*-glycan branching enzymes in mammals

**DOI:** 10.1016/j.jbc.2024.107471

**Published:** 2024-06-13

**Authors:** Yasuhiko Kizuka

**Affiliations:** Institute for Glyco-core Research (iGCORE), Gifu University, Gifu, Japan

**Keywords:** core fucose, fucosyltransferase, FUT8, glycobiology, glycoprotein biosynthesis, glycosylation, glycosyltransferase, GnT (MGAT), *N*-linked glycosylation

## Abstract

Most proteins in the secretory pathway are glycosylated, and *N*-glycans are estimated to be attached to over 7000 proteins in humans. As structural variation of *N*-glycans critically regulates the functions of a particular glycoprotein, it is pivotal to understand how structural diversity of *N*-glycans is generated in cells. One of the major factors conferring structural variation of *N*-glycans is the variable number of *N*-acetylglucosamine branches. These branch structures are biosynthesized by dedicated glycosyltransferases, including GnT-III (MGAT3), GnT-IVa (MGAT4A), GnT-IVb (MGAT4B), GnT-V (MGAT5), and GnT-IX (GnT-Vb, MGAT5B). In addition, the presence or absence of core modification of *N*-glycans, namely, core fucose (included as an *N*-glycan branch in this manuscript), synthesized by FUT8, also confers large structural variation on *N*-glycans, thereby crucially regulating many protein–protein interactions. Numerous biochemical and medical studies have revealed that these branch structures are involved in a wide range of physiological and pathological processes. However, the mechanisms regulating the activity of the biosynthetic glycosyltransferases are yet to be fully elucidated. In this review, we summarize the previous findings and recent updates regarding regulation of the activity of these *N*-glycan branching enzymes. We hope that such information will help readers to develop a comprehensive overview of the complex system regulating mammalian *N*-glycan maturation.

Protein glycosylation is one of the most frequent and abundant protein modifications in mammals, which regulates the functions of modified proteins in many aspects, such as folding, trafficking, activity, molecular interaction, and degradation (1, 2). The structural diversity of glycans is also huge. Glycans on proteins are basically classified into six groups, namely, *N*-glycan, *O*-glycan (further divided into several subclasses), glycosaminoglycan, glycosylphosphatidylinositol anchor, *C*-mannose, and nucleocytoplasmic *O*-GlcNAc (3). Among them, *N*-glycans are estimated to be attached to over 7000 proteins in humans (4), and the structural profile of *N*-glycans is completely different among cells, proteins, and even glycosylation sites. Since a subtle difference in *N*-glycan structure in a particular protein may result in significantly different biological effects and disease pathology (*e.g.*, cancer, dementia, diabetes, and COVID-19) (5, 6, 7, 8, 9), there is a need to clarify how *N*-glycan structures are maintained and become different in cells in order to obtain a deep understanding of the pathological mechanisms of these diseases.

When *N*-glycosylation is first initiated in the ER, the assembled oligosaccharide (Glc_3_Man_9_GlcNAc_2_) is first transferred to a nascent protein by oligosaccharyltransferase (OST) complex in mammals (Fig. 1*A*) (10). In the ER, during trimming of the terminal glucose (Glc) and mannose (Man) residues, *N*-glycans play essential roles in protein quality control, in which glycoproteins acquire correct folding, and misfolded glycoproteins are retrotranslocated to the cytosol and degraded by proteasomes. These quality control processes are mediated by specific interaction between lectins and *N*-glycan bearing a particular terminal structure. Subsequently, the folded proteins are transported to the Golgi where further glycan remodeling occurs *via* the orchestrated actions of many glycosidases/glycosyltransferases (Fig. 1*A*) (3). These enzymes sequentially and competitively act on *N*-glycans to shape their final complex structures, and the glycoproteins decorated with final *N*-glycan structures are trafficked to their destinations where they exert biological functions. Considering that diverse *N*-glycan structures are synthesized by complex actions of glycosyltransferases, it is pivotal to elucidate how each glycosyltransferase activity is regulated in cells.

**Figure 1:**
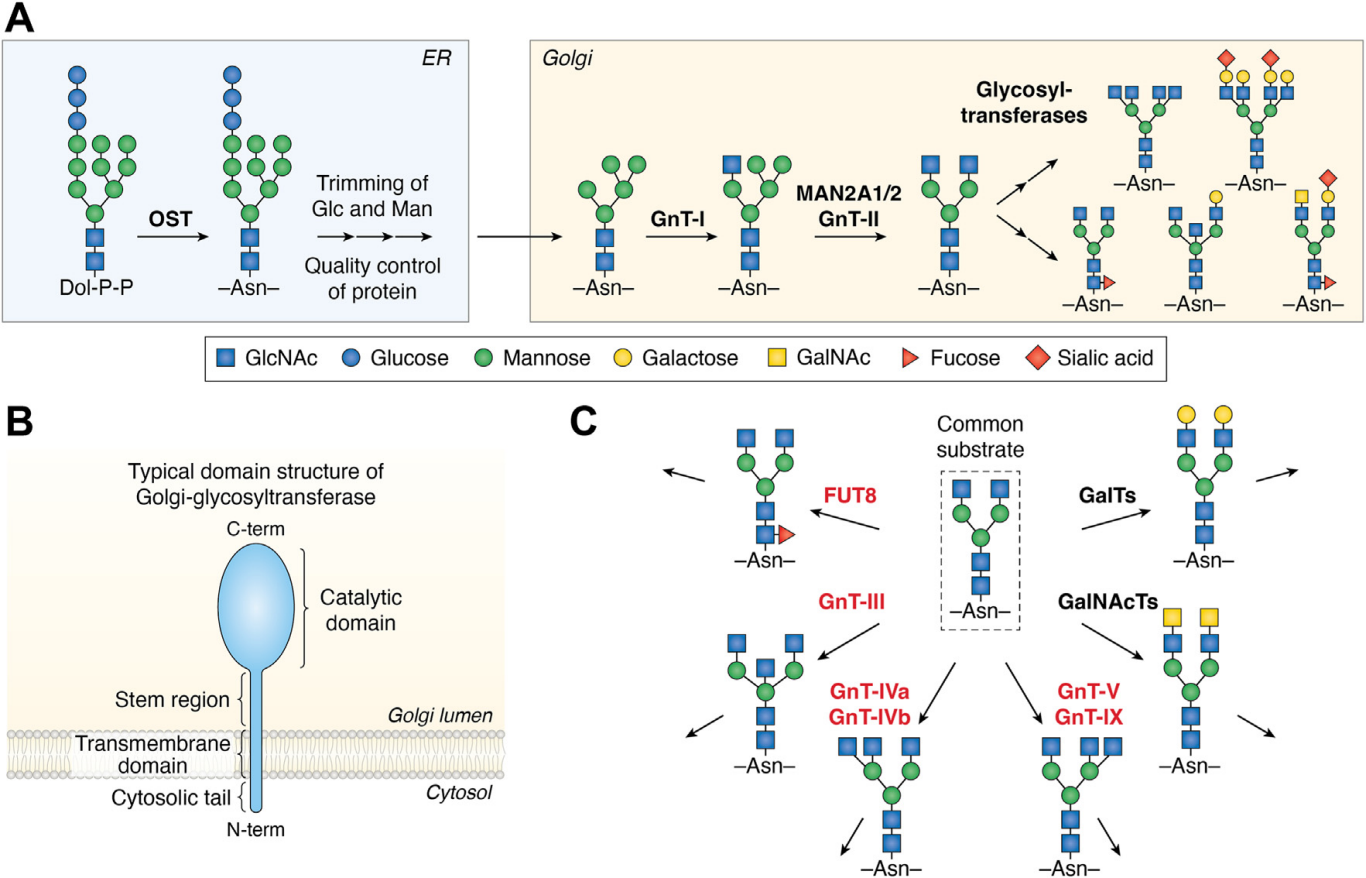
***N*-Glycan biosynthesis and related glycosyltransferases**. *A*, schematic drawing of *N*-glycan biosynthesis in the ER and Golgi apparatus. *B*, typical domain organization of Golgi-resident glycosyltransferases. These enzymes are type 2 membrane proteins having a short N-terminal cytosolic tail, transmembrane domain, stem region, and large C-terminal catalytic domain. *C*, basic pathways of *N*-glycan branching. A biantennary GlcNAc-terminated *N*-glycan is the common substrate for many glycosyltransferases, including branching enzymes. GlcNAc, *N*-acetylglucosamine; OST, oligosaccharyltransferase.

In general, Golgi-resident glycosyltransferases are single-pass type 2 transmembrane proteins that comprise a short N-terminal cytosolic tail, a transmembrane domain, a stem region, and a large C-terminal region containing a catalytic domain (Fig. 1*B*) (11). The activity of Golgi-resident glycosyltransferases in cells is likely regulated by various factors, including their gene expression, sub-Golgi localization, dimerization, shedding, heterocomplex formation, and degradation (12, 13, 14). However, these regulatory factors have not been sufficiently examined for each Golgi-resident glycosyltransferase.

During *N*-glycan maturation in the Golgi, the formation of a variable number of branches greatly contributes to increasing the structural complexity of *N*-glycans (15). After *N*-glycosylated proteins enter the cis-Golgi, the terminal Man residues are further trimmed by mannosidases to form a Man5 structure (Fig. 1*A*) (16). Subsequently, GnT-I (MGAT1) transfers *N*-acetylglucosamine (GlcNAc) to the Man5 structure to start the biosynthesis of complex-type *N*-glycans. After the Man trimming by MAN2A1 and MAN2A2, GnT-II (MGAT2) adds GlcNAc to form a common biantennary *N*-glycan (Fig. 1, *A* and *C*) (17). This glycan is a common substrate for various glycosyltransferases, including GlcNAc transferases, galactose (Gal) transferases, and GalNAc transferases (Fig. 1*C*). The GlcNAc transferases, GnT-III (MGAT3), GnT-IVa (MGAT4A), GnT-IVb (MGAT4B), GnT-V (MGAT5), and GnT-Vb (MGAT5B), form the respective branches in humans (18). Notably, in some cases, prior formation of one branch inhibits the formation of another branch, as described below, indicating that the reaction order of glycosyltransferases is important for shaping appropriate *N*-glycan structures in cells. The formed GlcNAc branches, with the exception of the GnT-III product, are further extended and capped with other monosaccharides. In addition, the core region of these GlcNAc-terminated *N*-glycans can be modified by a fucosyltransferase FUT8 with a fucose (Fuc) residue (Fig. 1*C*), which further adds structural complexity to *N*-glycans. Many previous studies using overexpression or knockout (KO) of each enzyme or clinical samples revealed that each branch is profoundly involved in the development and aggravation of various diseases, such as cancer and dementia, *via* regulation of the functions of specific glycoproteins as described below (15). These lines of accumulating evidence demonstrated that a particular branch or its biosynthetic enzyme could be a target for diagnosis and therapy for various diseases. However, how the activity of these enzymes is regulated in cells is not fully clear.

In this review, we describe the details of previous and recent findings regarding the regulation of activity of these *N*-glycan branching enzymes. In particular, we highlight the novel mechanisms and unanswered questions regarding their selectivity for protein substrates and the roles of noncatalytic domains. We hope that this review will inspire new research on exploring the biosynthetic mechanisms for complex *N*-glycans and their therapeutic and biotechnological applications.

## Bisecting GlcNAc synthesized by GnT-III (MGAT3) is a key residue for shaping *N*-glycan profile

**Figure 2:**
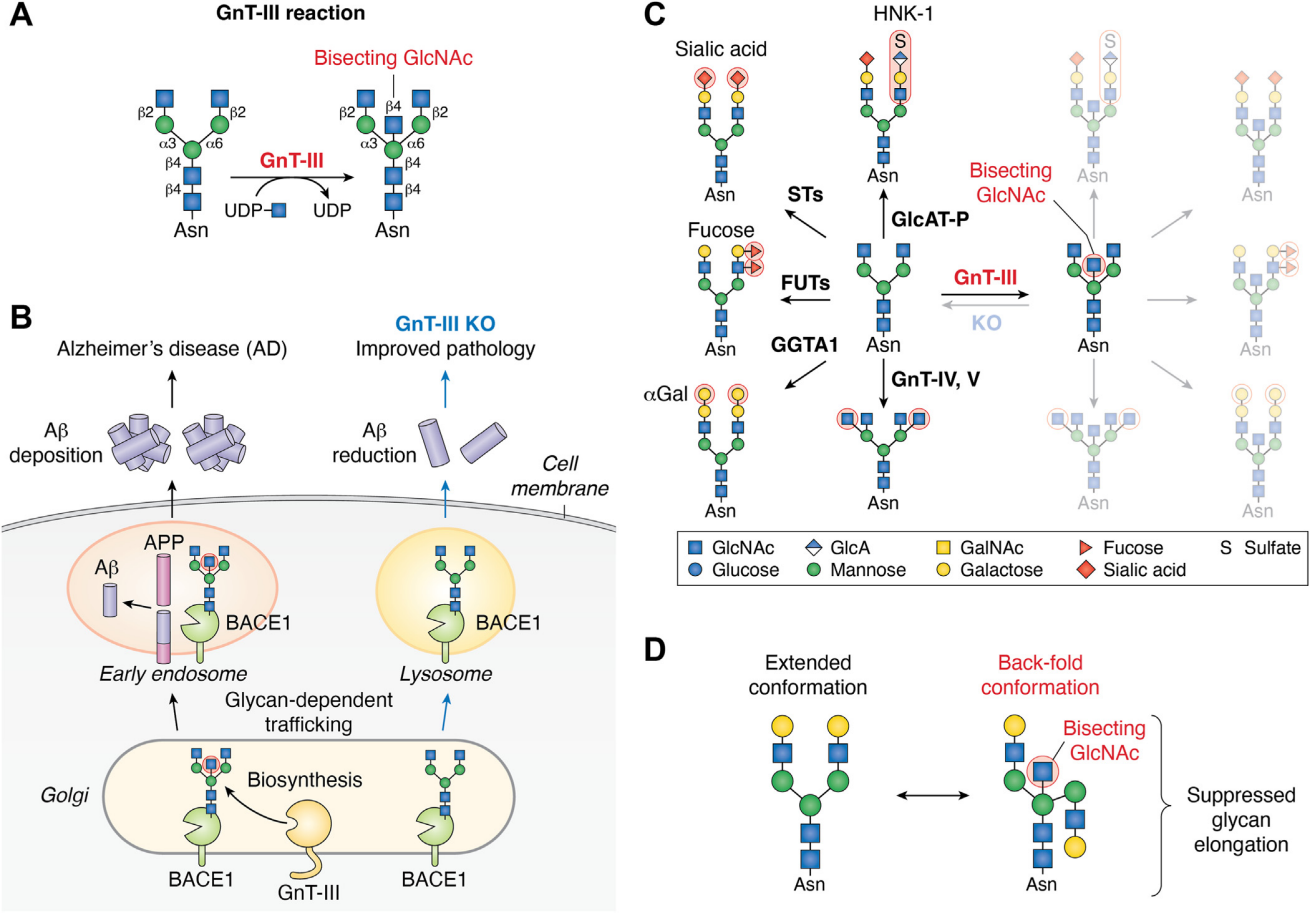
**Reaction and biological functions of GnT-III**. *A*, schematic drawing of the GnT-III reaction. *B*, a model for the improved Alzheimer’s disease pathology in GnT-III KO mice. The lack of bisecting GlcNAc on BACE1 led to aberrant targeting of BACE1 to the lysosomes and reduced production of Aβ peptide. *C*, the presence of bisecting GlcNAc suppresses the formation of other branches and terminal modifications of *N*-glycan. *D*, preferred conformation of nonbisected and bisected *N*-glycans. Bisected glycans preferentially adopt the back-fold conformation. GlcNAc, *N*-acetylglucosamine; KO, knockout.

GnT-III catalyzes the formation of a unique β1,4-GlcNAc branch on β-Man, designated as bisecting GlcNAc (Fig. 2*A*) (19). This enzyme is expressed in limited tissues, with the highest expression in brain (20), and indeed brain includes an abundance of bisecting GlcNAc-containing *N*-glycans (21, 22). Regarding the functions of this enzyme, GnT-III-KO mice showed significant improvement of Alzheimer’s disease pathology with reduced deposition of disease-causative Aβ peptide in brain (Fig. 2*B*) (5, 23, 24). This phenotype was found to be caused by aberrant lysosomal localization of the Aβ-producing enzyme BACE1 (5), suggesting that bisecting GlcNAc is involved in glycoprotein trafficking among trans-Golgi, endosomes, and lysosomes. Other studies showed that bisecting GlcNAc is also involved in the suppression of cancer pathology. The overexpression of GnT-III in B16 melanoma cells reduced metastasis to the lung in mice (25, 26). It was suggested that this reduced metastasis was caused by functional alterations of E-cadherin by bisecting GlcNAc modification and suppression of epithelial–mesenchymal transition by GnT-III (27, 28). Furthermore, GnT-III-KO mice showed an increased burden of mammary tumor in a polyoma virus middle T antigen–induced model (29, 30). These findings underscore the functional importance of this branch in various biological processes.

A unique feature of bisecting GlcNAc is the ability to inhibit further branching and terminal modifications in *N*-glycans. Many reports on *in vitro* enzyme activity of glycosyltransferases revealed that *N*-glycans bearing bisecting GlcNAc are not acceptable or poor substrates for many glycosyltransferases, including other GlcNAc-branching enzymes, GnT-IVa,b (31, 32) and GnT-V (33), core fucosylation enzyme FUT8 (34), and the enzymes acting on *N*-glycan terminals, including alpha-Gal transferase (35), sialyltransferases, fucosyltransferases, and HNK-1-synthesizing enzyme (36). This indicates that bisecting GlcNAc is a general suppressor of terminal modifications of *N*-glycans (Fig. 2*C*). In cells and tissues, the overexpression of GnT-III downregulated the expression of these glycan structures (35, 36), while the deletion of GnT-III resulted in an increase of these structures (36). Mechanistically, structural studies showed that the presence of bisecting GlcNAc enabled *N*-glycans to adopt a “back-fold” 3D structure as the preferred conformation (Fig. 2*D*) (36, 37, 38, 39), which can explain why bisected glycans are poor substrates for particular enzymes.

Regarding factors regulating GnT-III activity in cells, it was reported that GnT-III forms a heterocomplex with caveolin-1 (40). In terms of the significance of this complex formation, it was suggested to regulate the Golgi localization of GnT-III. As future work, it is important to search for other binding partners for GnT-III. Another important point is to clarify the selectivity for substrate proteins of GnT-III. Using wildtype (WT) and GnT-III KO mouse brains, we recently identified the glycoproteins and glycosites that are physiologically modified by GnT-III *in vivo* (41). However, to date, no common polypeptide motif for modification by GnT-III has been found, and it remains unclear how GnT-III selects its target glycoproteins in the Golgi. Solving the 3D structure of GnT-III could provide clues to clarify these issues.

## A unique lectin domain-assisted action of GnT-IVa,b (MGAT4A,B)

**Figure 3:**
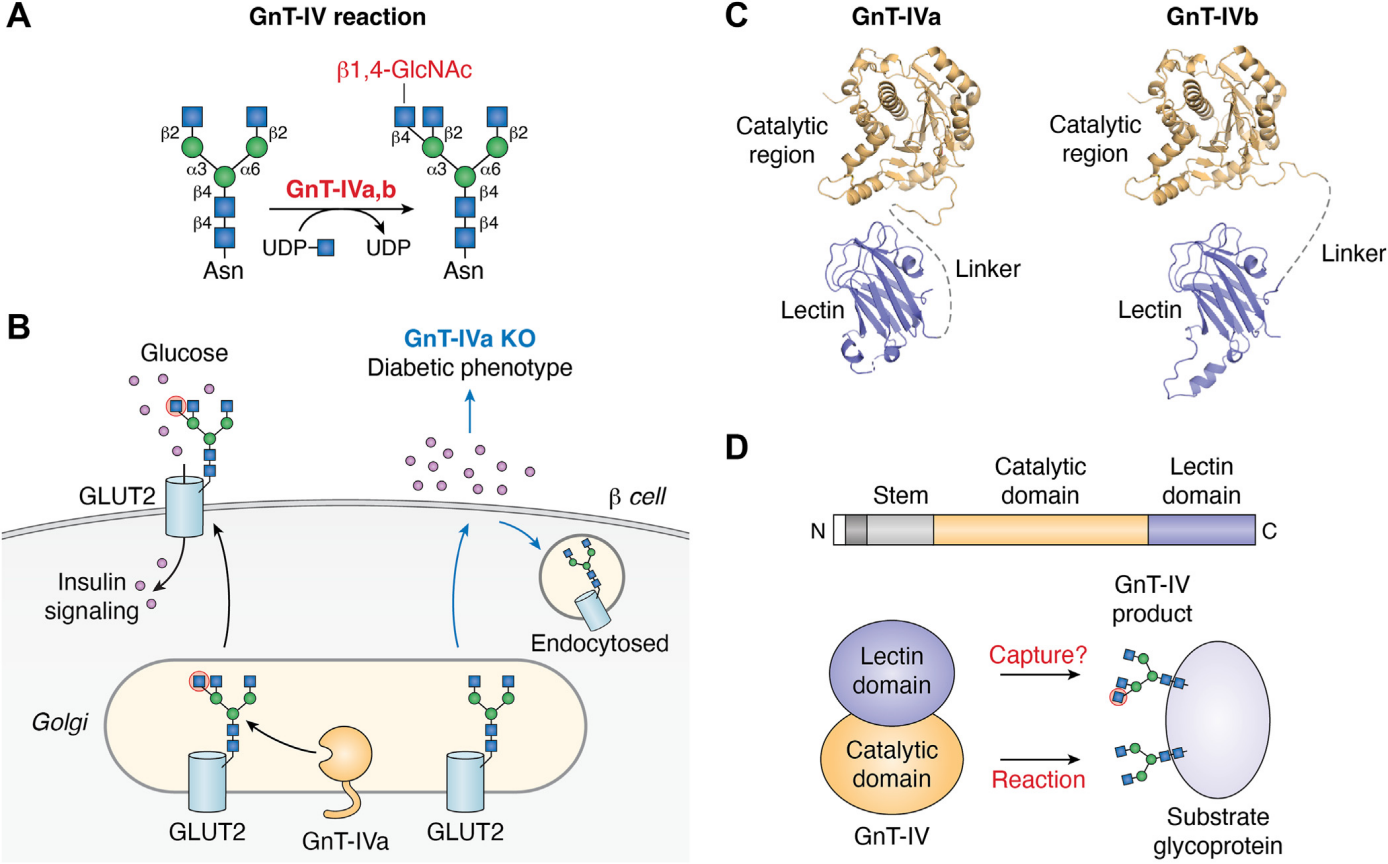
**Reaction and biological functions of GnT-IV**. *A*, schematic drawing of the GnT-IV reaction. *B*, a model for the type 2 diabetic phenotype in GnT-IVa KO mice. *C*, predicted structures of human GnT-IVa and GnT-IVb. The lectin domain of GnT-IVa is derived from the crystal structure of mouse GnT-IVa lectin domain (PDB ID: 7VMT). The two enzymes show very high structural similarity. *D*, domain structure of full-length GnT-IVa and GnT-IVb, and a model for the recognition of substrate glycoproteins by GnT-IVs *via* the lectin domain.

GnT-IVa and GnT-IVb catalyze the formation of a β1,4-GlcNAc branch on the α1,3-Man arm (Fig. 3*A*) (31, 42). Both GnT-IVa and GnT-IVb are enzymatically active *in vitro*, and it was shown that single KO of GnT-IVa partially reduced β1,4-GlcNAc branching, while double KO of GnT-IVa and GnT-IVb led to complete loss of GnT-IV activity as well as glycans with β1,4-branch in cells and tissues (43, 44). This demonstrated that both enzymes actively biosynthesize the branch. As for the biological functions of these enzymes, GnT-IVa-KO mice were found to exhibit type 2 diabetic phenotypes (45), such as hyperglycemia and impaired Glc-stimulated insulin secretion. This was shown to be caused by aberrantly enhanced internalization of glucose transporter 2, a key glycoprotein modified by GnT-IVa, in mouse pancreatic β cells (Fig. 3*B*). These findings suggested that functional modification of glucose transporter 2 by GnT-IVa is critical to Glc homeostasis. Moreover, uptake of a high-fat diet in mice reduced expression of the *Mgat4a* gene, and human diabetes patients also exhibited lower expression of *MGAT4A* mRNA (6), further demonstrating the involvement of GnT-IVa in the development of type 2 diabetes.

GnT-IVa and GnT-IVb show high sequence and structural homology (Fig. 3*C*). Although GnT-IVb exerts enzyme activity generating the same branch as GnT-IVa and shows almost ubiquitous tissue distribution, no overt phenotype has so far been found in GnT-IVb-KO mice (43). However, as described above, both GnT-IVa and GnT-IVb actively biosynthesize the β1,4-GlcNAc branch. Moreover, GnT-IVa and GnT-IVb were reported to be differentially involved in cancer malignancy and chemoresistance (46, 47). In addition, as described below, these two isozymes show different selectivity for glycoprotein substrates *in vitro*. These findings suggest that GnT-IVa and GnT-IVb have different biological functions.

Recently, we discovered that mouse GnT-IVa has a unique lectin domain at its C terminus and solved the 3D structure of this domain (48). As lectin domain deletion mutants completely lost the enzymatic activity (44), the lectin domain appears to be essential for GnT-IV’s functions. Given that this lectin domain is found in both GnT-IVa and GnT-IVb and that no other glycosyltransferases acting on *N*-glycans are known to have a lectin domain, we consider that the GnT-IV family has a unique reaction mechanism. A comprehensive binding assay using 157 glycans revealed that the GnT-IVa lectin domain specifically binds to GlcNAc-terminated *N*-glycans bearing the β1,4-GlcNAc branch, demonstrating that the lectin domain preferentially binds to the product glycans of the GnT-IV catalytic reaction (48). Another group also reported the crystal structure of the lectin domain of silkworm GnT-IV in complex with GlcNAc (49), clearly showing that the GnT-IV lectin domain is a GlcNAc-binding lectin, consistent with our report. As to the biological significance of recognition of glycans by the lectin domain, we found that the lectin domain is involved in determining the preference of glycoprotein substrates for GnT-IVa and GnT-IVb. We recently established *in vitro* enzyme assays for GnTs using UDP-Glo and several purified human serum glycoproteins (44, 50, 51). Using this system, we found that the preferred glycoprotein substrates differ between GnT-IVa and GnT-IVb and that GnT-IVb particularly prefers glycoproteins that had been partially premodified with a β1,4-GlcNAc branch (44). Considering that the lectin domain selectively binds to *N*-glycans bearing a β1,4-GlcNAc branch, these findings suggest that GnT-IVs interact with substrate glycoproteins by recognizing their glycans *via* the lectin domain (Fig. 3*D*). Consistent with this notion, replacement of a single amino acid in the GnT-IVb lectin domain altered its substrate selectivity to resemble that of GnT-IVa (44). These findings indicate that the lectin domain functions to select substrate glycoproteins for GnT-IVs.

Interestingly, the human GnT-IV family consists of four members: GnT-IVa (MGAT4A), GnT-IVb (MGAT4B), GnT-IVc (MGAT4C), and GnT-IVd (MGAT4D). In contrast to the case for GnT-IVa and GnT-IVb, the enzyme activity of human GnT-IVc and GnT-IVd has not been detected (52, 53). In birds and fish, GnT-IVc has a different branching activity, that is, GnT-VI activity, generating a β1,4-GlcNAc branch in the α1,6-Man arm (54, 55). Although the functions of this nonhuman-type branch are unclear, overexpression of chicken GnT-IVc in mammalian cells resulted in both aberrant formation of the *N*-glycan branch and remodeling of the intracellular metabolism of small molecules, such as amino acids (56). Clarifying the biological functions of GnT-IVc in humans is an interesting goal to pursue in future work. GnT-IVd lacks a C-terminal lectin domain, and no enzymatic activity of it has been found. This protein is also designated as GnT-I inhibitory protein and was found to inhibit GnT-I action and biosynthesis of complex-type *N*-glycans in cells (53, 57). Furthermore, GnT-IVd is specifically expressed in testis, and GnT-IVd-KO mouse testicular germ cells were found to be vulnerable to mild heat stress (58). These findings suggest that GnT-IVd-mediated suppression of *N*-glycan maturation regulates reproductive functions in testis, although GnT-IVd-KO male mice are fertile (58).

## Protein-selective modification by GnT-V (MGAT5) is mediated by its N domain

**Figure 4:**
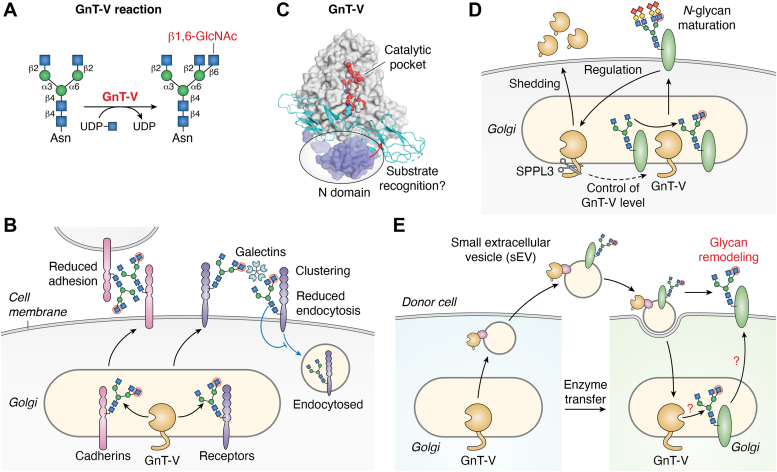
**Reaction and biological functions of GnT-V**. *A*, schematic drawing of the GnT-V reaction. *B*, a model for the functional regulation of cell adhesion molecules and cell surface receptors by GnT-V. The adhesive function of E-cadherin was reported to be suppressed by *N*-glycan branching by GnT-V, and molecular clustering or residency of the receptors at the cell surface is enhanced by galectin binding to the branched *N*-glycans synthesized by GnT-V. *C*, a docking model of human GnT-V and a glycoprotein substrate (E-cadherin). The N domain is suggested to be involved in recognizing polypeptides of substrate glycoproteins. *D*, a model for the shedding of GnT-V. SPPL3-mediated shedding of GnT-V is negatively regulated by blocking *N*-glycan maturation. *E*, a model for sEV-mediated cell-to-cell transfer of active GnT-V. A cleaved soluble form of GnT-V is loaded to the surface of sEVs and incorporated by another cell. How the soluble form of GnT-V can be associated with the surface of sEVs remains unclarified. sEVs, small extracellular vesicles.

GnT-V catalyzes β1,6-GlcNAc branching on the α1,6-Man arm (Fig. 4*A*) (33, 59). GnT-V has long been studied regarding its relationship with cancer development and metastasis (60). mRNA expression of *MGAT5* is upregulated by the oncogenic Ras-Raf-Ets pathway in various cancer cells (61, 62), and higher expression of GnT-V is associated with poor prognosis of cancer patients (63, 64). GnT-V functionally modifies cell adhesion molecules, including E-cadherin, and growth factor receptors, thereby promoting cancer cell migration and proliferation (65). A specific *N*-glycosylation site in E-cadherin was found to be modified by GnT-V, which was shown to suppress E-cadherin cis-dimerization and cell adhesion (66). Moreover, GnT-V-mediated biosynthesis of highly branched *N*-glycans on cell surface receptors led to prolonged residency of the receptors at the cell surface or induced molecular clustering at the cell surface (67, 68). These phenotypes were found to be mediated by increased interaction between branched *N*-glycans and galectins (Fig. 4*B*) (69). Consistent with the cancer-promoting roles of GnT-V, GnT-V-KO mice showed significantly reduced development and metastasis of polyoma virus middle T antigen–induced cancer (7), indicating that the inhibition of GnT-V activity is a potential new therapeutic option against cancer. Meanwhile, GnT-V-KO mice showed autoimmune phenotypes, which were postulated to be caused by hyperactivation of T-cell receptors (Fig. 4*B*) (67, 70). These findings suggest that compounds regulating GnT-V activity are candidates as therapeutic agents for cancer and autoimmune diseases.

To design specific inhibitors or activators of GnT-V and to elucidate the catalytic mechanism, we solved the crystal structure of human GnT-V luminal domain in complex with a bi-substrate-type inhibitor (71). This enabled identification of the catalytic center (E297 residue), and the specificity toward glycan substrates reported in biochemical studies was well explained by the 3D structure of the catalytic pocket. For instance, a bisected-type *N*-glycan, α1,3-Man arm, and extended α1,6-Man arm with Gal, neither of which is a substrate for GnT-V, do not fit the catalytic pocket and would cause steric hindrance (71). Another group also reported the crystal structure of the same luminal domain of GnT-V in complex with an acceptor pentasaccharide, further clarifying the interactions between the catalytic pocket and the donor and acceptor substrates (72).

Interestingly, our docking model of GnT-V with a longer acceptor substrate bearing the core region (chitobiose and Asn) suggested that GnT-V also interacts with the core region of the acceptor. This was supported by our MD simulation and biochemical experiments using various point mutants (51, 73). These findings raised the possibility that GnT-V recognizes the polypeptide part of acceptor glycoproteins. To explore this possibility, we focused on a unique domain (designated the N domain), which is located at the N-terminal side in the luminal region and could interact with the polypeptide moiety of acceptor proteins (Fig. 4*C*). Using a deletion mutant lacking the entire N domain and UDP-Glo assay as described above, we compared the activities of GnT-V and its ΔN mutant toward oligosaccharides, a glycopeptide, and glycoproteins. Surprisingly, ΔN mutant displayed large reductions in activity toward all glycoproteins tested, whereas ΔN mutant almost fully maintained its activity toward the oligosaccharides and the small glycopeptide (50). These findings demonstrate that the N domain is essential for activity toward glycoproteins, but not toward small substrates, suggesting that the N domain recognizes the polypeptide part of acceptor substrates (Fig. 4*C*). In this context, an important further issue is how the N domain is involved in recognizing polypeptide moieties of acceptor glycoproteins.

Intracellular activity and the protein level of GnT-V in cells were found to be regulated by the cleavage and secretion of GnT-V. A multispanning membrane protease, SPPL3, was identified as a major sheddase for various glycosyltransferases, including GnT-V (74). Silencing or KO of SPPL3 resulted in almost complete disappearance of the secreted GnT-V in the medium, while cellular GnT-V accumulated. In contrast, the overexpression of SPPL3 led to an increase in the secreted GnT-V and a decrease in cellular GnT-V, resulting in reduced *N*-glycan branching in the cells (Fig. 4*D*) (75). These findings clearly indicated that SPPL3-mediated shedding plays key roles in the regulation of cellular GnT-V activity. As SPPL3-mediated cleavage was reported to regulate both the cellular activity of various glycosyltransferases and the cellular glycome (76, 77), shedding of glycosyltransferases is a pivotal factor for glycosylation. Furthermore, we recently found that the SPPL3-mediated GnT-V shedding depends on the maturity of cellular *N*-glycans. When *N*-glycan maturation is blocked, GnT-V shedding is suppressed, and the protein level and activity of GnT-V in cells are upregulated (78). Such suppression of GnT-V shedding was observed upon the blocking of sialylation, galactosylation, or formation of complex *N*-glycans, suggesting that the loss of complex *N*-glycan structures with sialic acid inhibits the cleavage of GnT-V by SPPL3 (Fig. 4*D*). The biological significance of the regulation of GnT-V secretion and the mechanism of glycan structure-dependent shedding should be further explored in future work.

We also found that some of the GnT-V activity in cell culture media are also included in small extracellular vesicles (sEVs) derived from various cancer cells (79). sEVs, such as exosomes, are small vesicles secreted from all cells and contain various types of bioactive cargo, such as miRNA and glycoproteins (80, 81, 82). Glycosyltransferase activity assay for *N*-glycan branching enzymes revealed that GnT-V is selectively enriched in sEVs (79). Intriguingly, GnT-V in sEVs is a soluble form cleaved by SPPL3 and maintains its enzymatic activity. Furthermore, loading of GnT-V into sEVs was found to be suppressed by knocking out SPPL3 in cells, indicating that SPPL3-mediated cleavage is a prerequisite for the loading of GnT-V into sEVs. The protease protection assay and staining with membrane-impermeable fluorescent dye indicated that GnT-V resides at outer surface of sEV, suggesting that soluble GnT-V forms a complex with another molecule at the surface of sEV membrane. Surprisingly, the incubation of GnT-V KO cells with the GnT-V-containing sEVs restored the GnT-V activity in the recipient cells, as well as the expression of *N*-glycans bearing the GnT-V-produced branch (79). These findings indicate that active GnT-V can be transferred from a donor cell to a recipient cell *via* sEVs, where the transferred active GnT-V potentially acts as an active enzyme to locally produce the *N*-glycan branch (Fig. 4*E*). Such a nongenetic change in glycans is a novel mechanism for glycan remodeling in cells, and it would be intriguing to examine whether other glycosyltransferases are also present in sEVs and transferred to recipient cells.

## GnT-IX (GnT-Vb, MGAT5B), a GnT-V homolog acting on *O*-Man glycans *in vivo*

**Figure 5:**
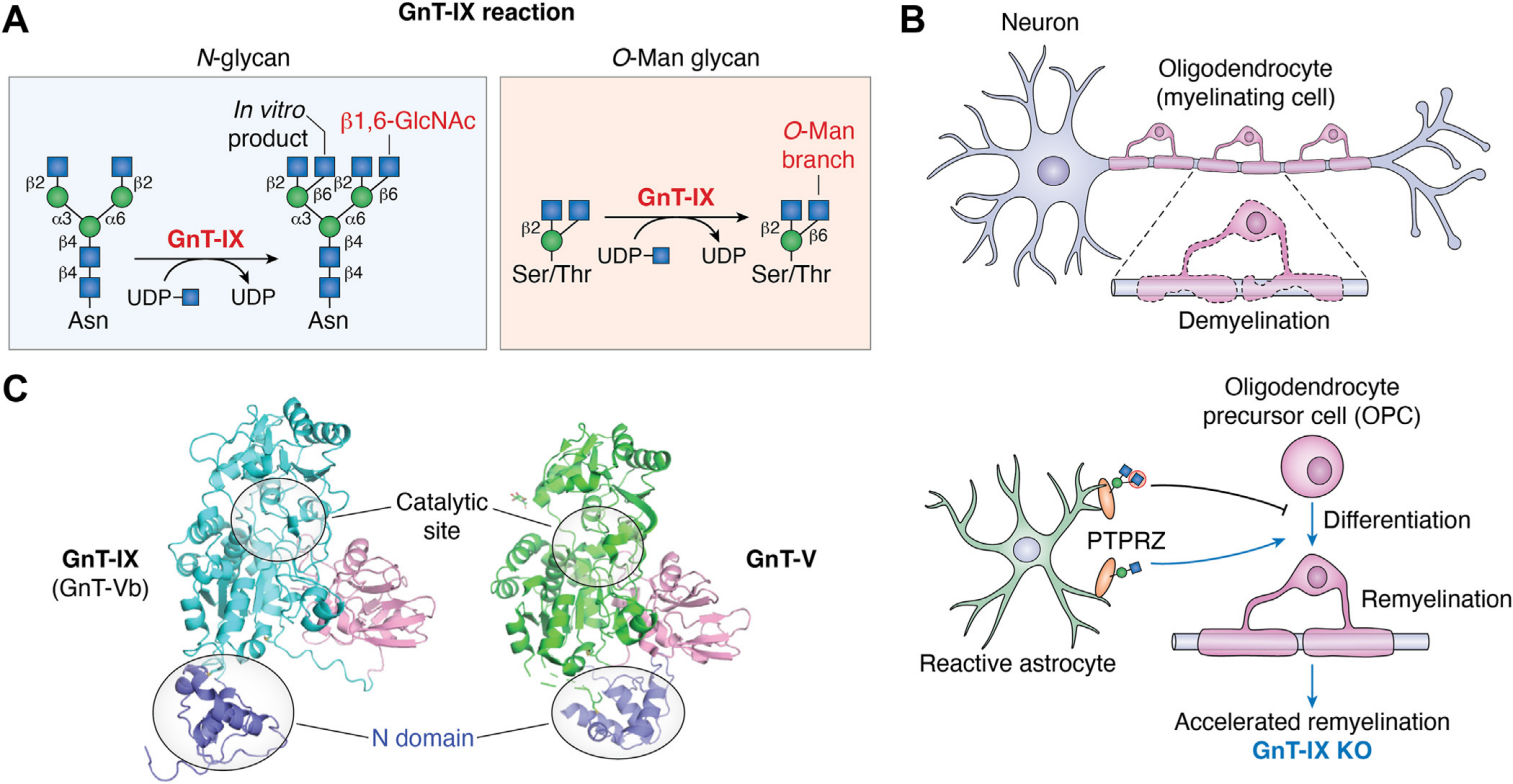
**Reaction and biological functions of GnT-IX (Vb).***A*, schematic drawing of the GnT-IX reaction. *B*, a model for the accelerated remyelination in GnT-IX KO mice. Branched *O*-Man glycans are synthesized by GnT-IX on PTPRZ in reactive astrocytes, which suppresses oligodendrocyte differentiation and remyelination. *C*, comparison of a predicted structure of human GnT-IX and the crystal structure of human GnT-V (PDB ID: 5ZIB). The structures of the N domain differ between the two enzymes. KO, knockout; PTPRZ, protein tyrosine phosphatase receptor type Z.

GnT-IX (GnT-Vb) was identified as a brain-specific homolog of GnT-V (83, 84). Although this enzyme showed the same branching activity on *N*-glycan as GnT-V (β1,6-branching on the α1,6-Man arm), it also showed another type of weak *N*-glycan branching activity to generate the β1,6-GlcNAc branch on the α1,3-Man arm only *in vitro* (Fig. 5*A*) (83). Later, in *in vitro* enzyme assays, it was revealed that GnT-IX highly preferentially acts on *O*-Man glycans to generate the β1,6-GlcNAc branch (85, 86), suggesting that GnT-IX functions as a branching enzyme for *O*-Man glycans *in vivo*. Consistent with this, in mice with the KO of GnT-IX, branched *O*-Man glycans almost completely disappeared in the brain (87), and in GnT-V-KO mouse brain, tetraantennary *N*-glycans were not detected (87). These findings revealed that GnT-IX is the branching enzyme for *O*-Man glycans.

Regarding the biological functions of GnT-IX, GnT-IX-producing branched *O*-Man glycans were reported to be involved in remyelination. Neuronal axons are myelinated by oligodendrocytes, which allows the rapid saltatory conduction of neurons (Fig. 5*B*). Acceleration of demyelination or impairment of remyelination causes demyelinating diseases, including multiple sclerosis (88). Branched *O*-Man glycans were reported to be attached to protein tyrosine phosphatase receptor type Z (PTPRZ) (89), and this glycoprotein was found to be involved in remyelination (90), raising the possibility that glycan modification of PTPRZ by GnT-IX could also be involved in remyelination. In fact, GnT-IX KO mice exhibited significantly improved remyelination after chemically induced demyelination with cuprizone (91). With regard to the mechanism involved, the accumulation in the damaged area of activated astrocytes positive for both PTPRZ and branched *O*-Man glycans in WT mouse brain was reduced in GnT-IX KO. As activated astrocytes were reported to suppress oligodendrocyte differentiation (92), these findings suggest that the formation of branched *O*-Man glycans on PTPRZ by GnT-IX is required for astrocyte activation, which in turn suppresses the generation of mature oligodendrocytes for remyelination (Fig. 5*B*). Recently, GnT-IX was also reported to be involved in astroglioma as well. GnT-IX KO glioma cells displayed significantly reduced growth in mouse brain (93), and this phenotype was suggested to be mediated by regulation of the protein level of PTPRZ by GnT-IX-mediated glycosylation. It remains unclear how GnT-IX is expressed in reactive astrocytes in demyelinating diseases and how GnT-IX is expressed in glioma cells, as GnT-IX is mainly expressed in neurons but not in isolated primary astrocytes from untreated WT mouse brain (94, 95).

Although the 3D structure of GnT-IX protein has not been solved yet, the AlphaFold-based predicted structure of GnT-IX is quite similar to that of GnT-V (Fig. 5*C*). In the future, structure-based study should reveal the structure and biochemical basis for the different acceptor specificities between GnT-V (for *N*-glycan) and GnT-IX (for *O*-Man glycan). In addition, GnT-IX also possesses an N domain, but its predicted structure is different from that of GnT-V (Fig. 5*C*), which might contribute to recognition of the substrate proteins by GnT-IX. So far, only limited glycoproteins have been found to be modified by GnT-IX with branched *O*-Man glycans, such as PTPRZ, CD24, and Neurofascin186 (91, 96, 97). Since it is unclear whether and how GnT-IX selectively modifies these glycoproteins, an important next step is to identify the specific physiological substrate proteins for GnT-IX. At present, no probe is available for the specific detection of branched *O*-Man glycans, namely, the GnT-IX products. There is thus a need to develop a new probe for GnT-IX-producing glycans for future study.

## Core Fuc synthesized by FUT8 is a common core structure of *N*-glycans

**Figure 6:**
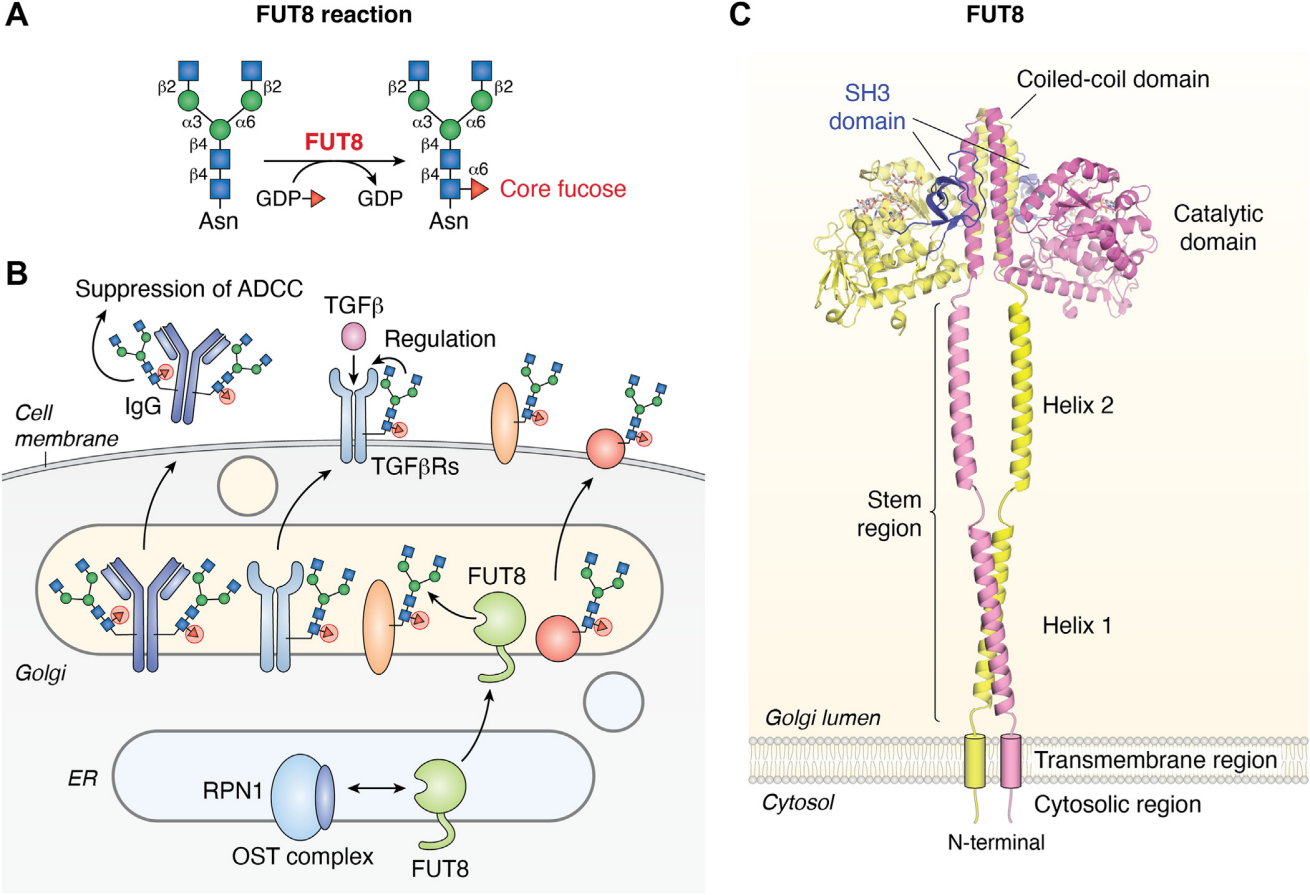
**Reaction and biological functions of FUT8**. *A*, schematic drawing of the FUT8 reaction. *B*, a model for the regulation of glycoprotein functions by FUT8. FUT8 modifies many glycoproteins in the Golgi apparatus, which in turn regulates many biological processes, including ADCC activity of therapeutic IgG and receptor–ligand interaction of TGFβ signaling. *C*, a predicted dimer structure of human FUT8. ADCC, antibody-dependent cellular cytotoxicity; OST, oligosaccharyltransferase; RPN1, ribophorin I; SH3, Src homology 3.

FUT8 catalyzes the addition of Fuc to the innermost GlcNAc residue to generate a core Fuc structure (Fig. 6*A*) (98, 99). Core Fuc is frequently attached to *N*-glycans in various tissues. For instance, over 90% of *N*-glycans from human serum IgG are core fucosylated (100). Furthermore, the functional significance of core Fuc has been revealed for many glycoproteins. Removal of core Fuc from therapeutic IgG was found to raise its antibody-dependent cellular cytotoxicity by ∼100-fold (101), so this approach is now used clinically. In addition, mice with FUT8 deficiency showed numerous phenotypes, including early death, growth retardation, emphysematous changes in lung, impaired T-cell functions, resistance to ulcerative colitis, and schizophrenia-like behaviors (102, 103, 104). In humans, FUT8 deficiency was reported to be one of the congenital disorders of glycosylation with severe symptoms, including developmental delays and respiratory abnormalities (105, 106). All of these findings demonstrated that FUT8-produced core Fuc is physiologically important. In addition to its physiological importance, core Fuc was also found to be involved in various diseases. For example, reduced core fucosylation in IgG was found to be correlated with COVID-19 severity (9), and core Fuc was also reported to promote melanoma metastasis (107) and therapeutic resistance of cancer (108). Although the precise mechanisms for all of these FUT8-related phenotypes and diseases have not been clarified, loss of core Fuc on several glycoproteins, such as TGFβ receptors and IgG, is known to affect their binding to their interaction partners (Fig. 6*B*) (102, 109).

FUT8 has unique domain organization, and the Src homology 3 (SH3) domain is found at its C terminus (Fig. 6*C*) (110, 111). The SH3 domain is basically involved in interaction with proline-rich regions (112) and is found only in FUT8 among human glycosyltransferases. This suggests that this domain plays a key role in FUT8 functions. We and Tomida *et al*. and Ihar*a et al.* found that a mutant lacking the SH3 domain or several point mutants in this domain barely possessed enzymatic activity *in vitro* (113, 114). In addition, recent structural studies of FUT8 crystals in complex with donor and acceptor substrates revealed that the SH3 domain interacts with the β1,2-GlcNAc residue on the α1,3-Man arm, which is the product of GnT-I (115, 116, 117). This is well consistent with the biochemical observation that FUT8 basically requires the β1,2-GlcNAc residue on the α1,3-Man arm in acceptor substrates (115, 118). However, it was also reported that FUT8 can act on oligomannose-type *N*-glycans attached to limited substrates even without the GlcNAc residue *in vitro* (119, 120), and indeed core Fuc was detected on oligomannosidic *N*-glycans derived from biological samples (121, 122). This demonstrated that FUT8 potentially modifies oligomannose *N*-glycans, at least for some proteins. Since the current FUT8 3D structure cannot simply explain how it accommodates oligomannose glycans without β1,2-GlcNAc, further structural and biochemical studies will be needed to resolve this issue.

We also found that expressed FUT8ΔSH3 mutants showed different subcellular localization from FUT8 WT in cells. FUT8 WT showed not only Golgi localization but also partial localization at the cell surface, whereas such partial staining at the cell surface was reduced in FUT8ΔSH3 mutants (113). Although the molecular mechanism for the subcellular trafficking of FUT8 is unclear, this suggests that the SH3 domain is also involved in subcellular trafficking of FUT8 as well as in the recognition of acceptor substrate glycans. Furthermore, our proteomic study identified ribophorin I (RPN1) as a FUT8-interacting protein (113). As FUT8ΔSH3 mutants showed weaker interaction with RPN1 than FUT8 WT did, the SH3 domain was suggested to be involved in this binding. Regarding the function of this interaction, knocking down RPN1 in cells led to a decrease in the activity of endogenous FUT8 (113), showing that RPN1 is a positive regulator of FUT8 activity. RPN1 is a subunit of the OST complex that is the initiation enzyme for *N*-glycosylation (Fig. 1*A*) (123, 124), and it remains unclear whether other OST subunits are also involved in the interaction with FUT8. Moreover, it is reasonable that OST subunits basically reside and function in the ER (125), which is different from the major subcellular localization of FUT8 in the Golgi. To fully understand the mechanisms regulating FUT8 activity in cells, it will be necessary to clarify where FUT8 interacts with RPN1 and how it positively regulates FUT8 activity.

We also recently focused on the FUT8 stem region and found that it is required for oligomerization (126). In general, glycosyltransferases have a stem region at the N terminus in a large luminal region, and this region is required for Golgi localization or multimerization of glycosyltransferases (11). Mutants lacking the FUT8 stem region showed impairment of oligomerization and resultant rapid degradation by proteasomes (126). The FUT8 stem region consists of two helices, and we found that the N-terminal helix plays a major role in FUT8 oligomerization (Fig. 6*C*). These findings suggest that the first helix-mediated multimerization of FUT8 is necessary for the formation of a stable FUT8 structure.

## Future perspectives

We here summarized the known biological functions, structure, and domain organization, substrate specificity, and activity regulation of *N*-glycan branching enzymes. Although several novel mechanisms of regulation of these enzymes have recently been reported, many issues remain to be clarified. For instance, the selectivity of substrate proteins of these enzymes is largely unknown, and identification of their physiological substrate proteins by nonbiased proteomics would be useful to clarify the selection mechanisms. Moreover, solving the 3D structures of these enzymes in complex with various substrates is also crucial. Elucidating the mechanisms regulating intracellular activity of *N*-glycan branching enzymes will include shedding, fine sub-Golgi localization, and formation of heterocomplex of endogenous enzymes in more detail. Clarifying these issues should contribute to understanding how complex *N*-glycans are biosynthesized in cells.

## Conflict of interest

The author declares that he has no conflicts of interest with regard to the contents of this article.
